# Comparative Performance of Electrode Models for Thickness-Shear AT-Cut Quartz Resonators

**DOI:** 10.3390/mi17091023

**Published:** 2026-08-28

**Authors:** Hang Chen, Xin Fu, Wanli Yang, Chao Zhan, Feng Xiong, Xiaohu Wang, Hongping Hu, Yuantai Hu

**Affiliations:** 1 Department of Engineering Mechanics, School of Aerospace Engineering, Huazhong University of Science and Technology, Wuhan 430074, China; m202472033@hust.edu.cn (H.C.); fux@hust.edu.cn (X.F.); huhp@hust.edu.cn (H.H.); hudeng@263.net (Y.H.); 2 Hubei Key Laboratory of Engineering Structural Analysis and Safety Assessment, Huazhong University of Science and Technology, Wuhan 430074, China; 3 TKD Science and Technology Co., Ltd., Suizhou 441300, China; zc@sztkd.com (C.Z.); xf@sztkd.com (F.X.); wxh@sztkd.com (X.W.); 4 Hubei Key Laboratory of Micro-Nano Crystal Processing Technology, TKD Science and Technology Co., Ltd., Suizhou 441300, China

**Keywords:** quartz resonator, electrode model, finite element analysis, cut angle, electrode mass

## Abstract

In this study, we compare four electrode models, i.e., solid model, added mass model, thin-film model, and a combining model of thin-film with added mass, for a 76.8 MHz AT-cut quartz resonator. Relative to the high-fidelity solid electrode benchmark, the combining model accurately captures the thickness-shear mode but overestimates displacement confinement due to mass loading and boundary effects. The thin-film model fails to replicate practical device states by ignoring mechanical mass and stiffness. In contrast, the added mass model optimally balances fidelity and efficiency, matching the benchmark’s frequency and resistance predictions with a 43% reduction in computation time. Parametric studies further indicate that cut angle deviations (33°~37°) alter equivalent shear stiffness, while electrode mass variations (mass coefficient 0.99~1.02) modify vibrational inertia. Both of them are critical for frequency tuning. Our findings clarify the scope and limitations of these strategies, guiding high-precision simulations and designs for high-frequency resonators.

## 1. Introduction

Due to its stable temperature frequency characteristics, high quality factor (*Q* factor), low phase noise and superior long-term frequency stability, quartz crystal resonators (QCRs) have long served as core components of frequency control and precision timing systems, and are widely deployed in wireless communication, navigation and positioning, aerospace, industrial control, micro/nano sensing and other fields [1,2,3,4,5,6]. With the rapid development of the fifth generation mobile communication (5G), Internet of Things (IoT), high-precision sensor networks, and miniaturized electronic systems, higher requirements are imposed on the miniaturization, frequency stability, and low power consumption of frequency devices, which makes the structural design and performance optimization of quartz resonators a research hotspot again [7,8,9]. Among various crystal cuts, the AT-cut quartz crystal stands out owing to its thickness-shear (TS) vibration mode and excellent frequency-temperature characteristics, which maintain low frequency drift within the industrial temperature range. Hence, AT-cut remains the most widely adopted cut type for commercial quartz resonators [10,11].

The overall performance of AT-cut quartz resonators is governed by the combined effects of the anisotropic elastic constants, piezoelectric constants and their temperature coefficients of quartz crystals. A tiny deviation in the cut angle of the wafer will alter remarkably the equivalent material parameters and frequency-temperature curves, thereby affecting the resonance frequency, dynamic resistance, Q factor and spurious mode distribution [12,13]. Meanwhile, as the operating frequency increases and device size shrinks continuously, traditional empirical design methods can no longer meet the demands for developing high-performance resonators. Consequently, establishing accurate theoretical models and numerical simulation methods has become an essential foundation for the design of quartz resonators.

The research model of quartz resonators has evolved from analytical models to numerical models. Mindlin plate theory and Tiersten piezoelectric plate theory provide the theoretical framework for analyzing the vibration of quartz plates [14,15]. Relevant studies have revealed the coupling mechanisms among thickness-shear mode, thickness-torsion mode, and bending mode, and have established the theoretical system of energy trapping. According to the energy trapping principle, the mass loading effect of electrodes reduces the cutoff frequency in the electrode coverage region, confines vibration energy within that area, and suppresses energy leakage towards supporting structures and boundaries [16,17,18]. This mechanism directly determines the mode purity and Q factor, and serves as a critical theoretical guideline for the design of modern quartz resonators.

Nevertheless, when the operating frequency extends to the high-frequency and ultra-high-frequency ranges, two-dimensional plate theories exhibit inherent limitations in handling electrode edge effects, local stress concentration, high-order mode coupling, and complex boundary conditions [19,20]. As a powerful numerical approach, the finite element method (FEM) can comprehensively take piezoelectric coupling, material anisotropy, electrode configuration, dielectric boundary conditions, and packaging constraints into account, and has become a dominant tool for the analysis and optimization of quartz resonators. In recent years, multi-physics simulation platforms including COMSOL Multiphysics, ANSYS and ABAQUS have been extensively applied to investigate the frequency response, admittance characteristics, frequency-temperature behavior, and energy trapping of quartz resonator [21,22,23,24]. Particularly for high-frequency and miniaturized resonators, three-dimensional finite element models can reproduce the actual vibration behaviors more realistically. In finite element modeling, the treatment of electrodes is a dominant factor affecting numerical accuracy [25,26].

The electrodes of the resonator not only undertake the functions of excitation and signal detection, but also introduce mass loads, stiffness variation, and local redistribution of electric field. The material, thickness, coverage area, and alignment error of upper and lower electrodes will all affect the resonance frequency and vibration mode. Currently, three mainstream electrode modeling approaches are widely used: the solid electrode model, the equivalent mass loading model and the thin-film electrode model. The solid electrode model can fully describe the internal stress and electric field distribution inside electrodes, but it requires enormous computational resources due to the small mesh size in the thickness direction. The equivalent mass loading model achieves high computational efficiency at the cost of neglecting electrode stiffness and interfacial coupling effects. The thin-film electrode model is applicable to the analysis of micro/nano-scale resonators, while its scope of application is restricted by electrode thickness and meshing strategies [27]. Although existing researches have discussed the effects of electrode material, shape, ring electrodes, multi-ring electrodes and transverse electric field excitation structures on vibration characteristics, systematic comparative studies on electrode modeling strategies are still insufficient. Different modeling methods may cause frequency deviations and mode errors with varying degrees, so it is necessary to systematically evaluate their applicability and computational accuracy [28,29,30,31].

In addition to electrode effect, cut angle deviation is another major factor deteriorating the performance of quartz resonators. The frequency-temperature characteristics of AT-cut quartz crystals are extremely sensitive to the cut angle. Even deviations of a few arc minutes or arc seconds during manufacturing process will lead to significant changes in the frequency-temperature curve [32,33]. When the primary vibration mode and adjacent spurious modes possess similar temperature drift rates, temperature variation may induce mode coupling, further resulting in some phenomena, such as frequency jumps, admittance peak splitting, abnormal increase of dynamic resistance, and so-called activity dip. In recent years, increasing attention has been paid to cut angle error, parallelism error, surface roughness, and contour machining error [13,34,35]. Existing investigations indicate that these non-ideal factors will break the symmetry of the thickness-shear mode, intensify spurious mode coupling, and thus degrade the frequency stability and Q factor of the devices.

In this study, the finite element models of AT-cut quartz crystal resonators are constructed with four electrode models, namely the solid electrode model, the added mass electrode model, thin-film electrode model and combining electrode model with thin-film and added mass. The resonance frequency, mode shape distribution, admittance response and energy trapping characteristics are compared and analyzed by different modeling methods. On this basis, the cut angle deviation parameter is introduced to explore the evolution law of frequency characteristics and modal behaviors with cut angle. Through the systematic comparison of simulation results under different electrode models and cut angle deviations, the applicable ranges and error sources of various electrode modeling methods are clarified. This work provides a theoretical basis for high-precision finite element modeling and structural optimization design of quartz resonators.

## 2. Materials and Models

### 2.1. Modeling and Material Parameters

The material parameters of quartz, including density, dielectric constants, elastic compliance, elastic stiffness, and piezoelectric constants, are presented. Since quartz is an anisotropic piezoelectric material, its constitutive relations require a full set of independent constants, which are provided below. 

The geometric dimensions of the resonator model are listed in Table 1.

**Figure 1:**
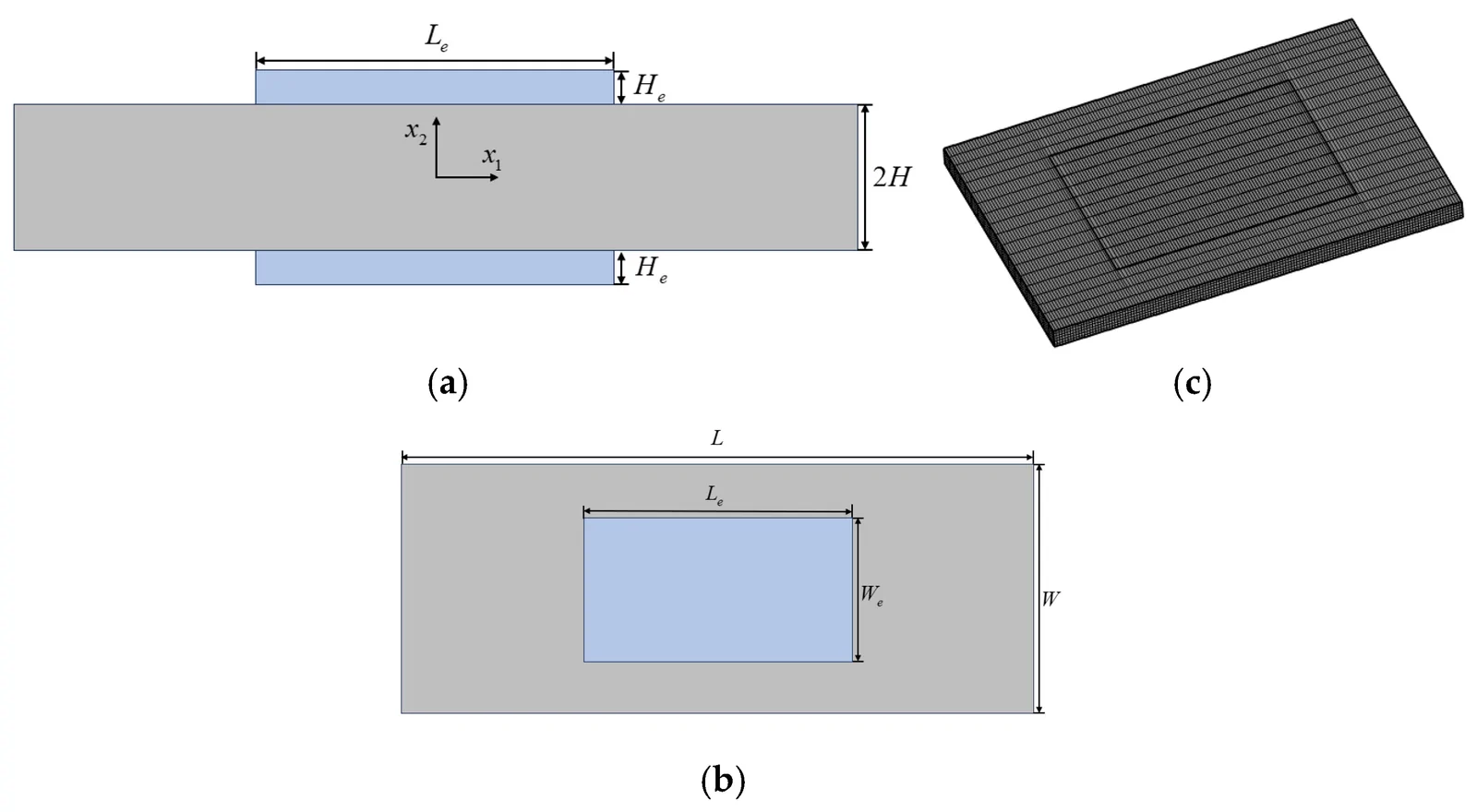
Model of the quartz resonator: (**a**) Plan view in the *x*_1_–*x*_2_ plane; (**b**) Plan view in the *x*_1_–*x*_3_ plane; (**c**) Computational mesh.

The geometric model and computational mesh of the quartz resonator are presented in Figure 1.

For the quartz, density $\rho =2651{\;\mathrm{kg}/m}^{3}$. The dielectric constant matrix is

(1) $$\varepsilon =\left(\begin{array}{ccc} \varepsilon_{11} & 0 & 0\\ 0 & \varepsilon_{22} & 0\\ 0 & 0 & \varepsilon_{33} \end{array}\right)=\left(\begin{array}{ccc} 4.514\varepsilon_{0} & 0 & 0\\ 0 & 4.514\varepsilon_{0} & 0\\ 0 & 0 & 4.634\varepsilon_{0} \end{array}\right)$$

where $\varepsilon_{0}=8.854\times 10^{-12}\;F/m$ is the permittivity of free space.

As a 32 point group crystal, the quartz has the following elastic compliance matrix **c** as

(2) $$c=\left[\begin{array}{cccccc} c_{11} & c_{12} & c_{13} & c_{14} & 0 & 0\\ c_{12} & c_{11} & c_{13} & -c_{14} & 0 & 0\\ c_{13} & c_{13} & c_{33} & 0 & 0 & 0\\ c_{14} & -c_{14} & 0 & c_{44} & 0 & 0\\ 0 & 0 & 0 & 0 & c_{44} & c_{14}\\ 0 & 0 & 0 & 0 & c_{14} & c_{66} \end{array}\right]$$

with the corresponding stiffness coefficients as

$$c_{11}=8.674\times 10^{10}{\;N/m}^{2},\;c_{12}=0.699\times 10^{10}{\;N/m}^{2},\;c_{13}=1.191\times 10^{10}{\;N/m}^{2},$$

$$c_{14}=1.791\times 10^{10}{\;N/m}^{2},\;c_{33}=10.719\times 10^{10}{\;N/m}^{2},\;c_{44}=5.794\times 10^{10}{\;N/m}^{2},$$

$$c_{66}=\frac{1}{2}(c_{11}-c_{12})=3.991\times 10^{10}{\;N/m}^{2}.$$

The piezoelectric constant matrix **e**, which couples the electric field with mechanical strain, is given for quartz (point group 32) as

(3) $$e=\left(\begin{array}{cccccc} -e_{11} & e_{11} & 0 & -e_{14} & 0 & 0\\ 0 & 0 & 0 & 0 & e_{14} & e_{11}\\ 0 & 0 & 0 & 0 & 0 & 0 \end{array}\right)$$

where $e_{11}=0.171{\;C/m}^{2}$ and $d_{14}=0.0406{\;C/m}^{2}$.

Moreover, for the gold electrodes, density, Young’s modulus, and Poisson’s ratio are $\rho_{e}=19,300{\;\mathrm{kg}/m}^{3}$, $E_{e}=70\;\mathrm{GPa}$, and $\mu_{e}=0.44$, respectively. The shear modulus $G_{e}=E_{e}/\left(1+\mu_{e}\right)$.

To account for the viscosity-induced loss of quartz, the elastic stiffness matrix was expressed in complex form as [36]

(4) $${\tilde{c}}_{pq}=c_{pq}+I\omega \eta_{pq}$$

where $\eta_{pq}(p,q=1,2,\ldots ,6)$ is the anisotropic viscosity-coefficient matrix. I is the imaginary unit, and ω=2πf is the angular frequency. Notably, the same complex stiffness matrix and viscosity coefficients were assigned to the quartz substrate in all four electrode models.

Besides, the *Q* factor was also calculated from the complex eigenfrequency as

(5) Q=Re(fc)2Im(fc)

where Re(fc) and Im(fc) are the real and imaginary parts of the complex eigenfrequency, respectively.

### 2.2. Theoretical Solution of Resonance Frequency

Based on one-dimensional quartz resonator model [37], only odd order modes can be excited. The odd order resonance frequency can be written as

(6) $$f_{i}=\frac{v\xi_{i}}{2\pi }$$

where, phase velocity of quartz $v=\sqrt{{\bar{c}}_{66}/\rho }$. ${\bar{c}}_{66}=c_{66}\left(1+k_{26}^{2}\right)$ with $k_{26}^{2}=e_{26}^{2}/\varepsilon_{22}c_{66}$. Wave numbers $\xi_{i}$ can be calculated from [37]

(7) $$\left(1-{\bar{k}}_{26}^{2}\frac{\tan\xi H}{\xi H}-\sqrt{Z_{r}}\tan\xi H\tan\xi_{e}H_{e}\right)\left(\tan\xi H+\sqrt{Z_{r}}\tan\xi_{e}H_{e}\right)=0$$

where ${\bar{k}}_{26}^{2}=k_{26}^{2}/\left(1+k_{26}^{2}\right)$. For the electrode, wave number $\xi_{e}=v_{e}\xi /v$ with phase velocity $v_{e}=\sqrt{G_{e}/\rho_{e}}$. The impedance ratio *Z_r_* of electrode metal and quartz is

(8) $$Z_{r}=\frac{\rho_{e}G_{e}}{\rho {\bar{c}}_{66}}$$

For a rotated Y-cut quartz, the rotation angle about the *x*-axis is denoted by ϕ. The coordinate transformation matrix **A** is

(9) $$A=\left[\begin{array}{ccc} 1 & 0 & 0\\ 0 & \cos\phi & -\sin\phi \\ 0 & \sin\phi & \cos\phi \end{array}\right]$$

where m=cosϕ and n=sinϕ. The Voigt strain transformation matrix of the transformed material is given as

(10) $$M=\left[\begin{array}{cccccc} 1 & 0 & 0 & 0 & 0 & 0\\ 0 & m^{2} & n^{2} & -2mn & 0 & 0\\ 0 & n^{2} & m^{2} & 2mn & 0 & 0\\ 0 & mn & -mn & m^{2}-n^{2} & 0 & 0\\ 0 & 0 & 0 & 0 & m & n\\ 0 & 0 & 0 & 0 & -n & m \end{array}\right]$$

Therefore, the elastic stiffness matrix after coordinate transformation can be rewritten as

(11) $$c^{\prime }=McM^{T}$$

The shear stiffness coefficient *c*_66_ then becomes

(12) $$c_{66}^{\prime }=c_{44}n^{2}+c_{66}m^{2}-2c_{14}mn.$$

$e_{26}$ and $\varepsilon_{22}$ are transformed as

(13) $$\begin{array}{l} \varepsilon_{22}^{\prime }=m^{2}\varepsilon_{11}+n^{2}\varepsilon_{33}\\ e_{26}^{\prime }=-e_{11}m^{2}-e_{14}mn \end{array}$$

The resonance frequencies of a Y cut quartz plate with any an angle can be obtained by Equations (5) and (6).

### 2.3. Solid Electrode Model

The solid electrode model employs three-dimensional solid elements to describe the geometric profile, thickness, and spatial distribution of the electrodes. This model comprehensively accounts for the mass, stiffness, and geometric boundary effects of the electrodes, thereby providing a relatively accurately representation of the electrodes on the vibration characteristics, energy localization, and electromechanical coupling behavior of the quartz crystal. Therefore, the solid electrode model is a modeling method that can accurately describe the working behavior of quartz resonator electrodes.

**Figure 2:**
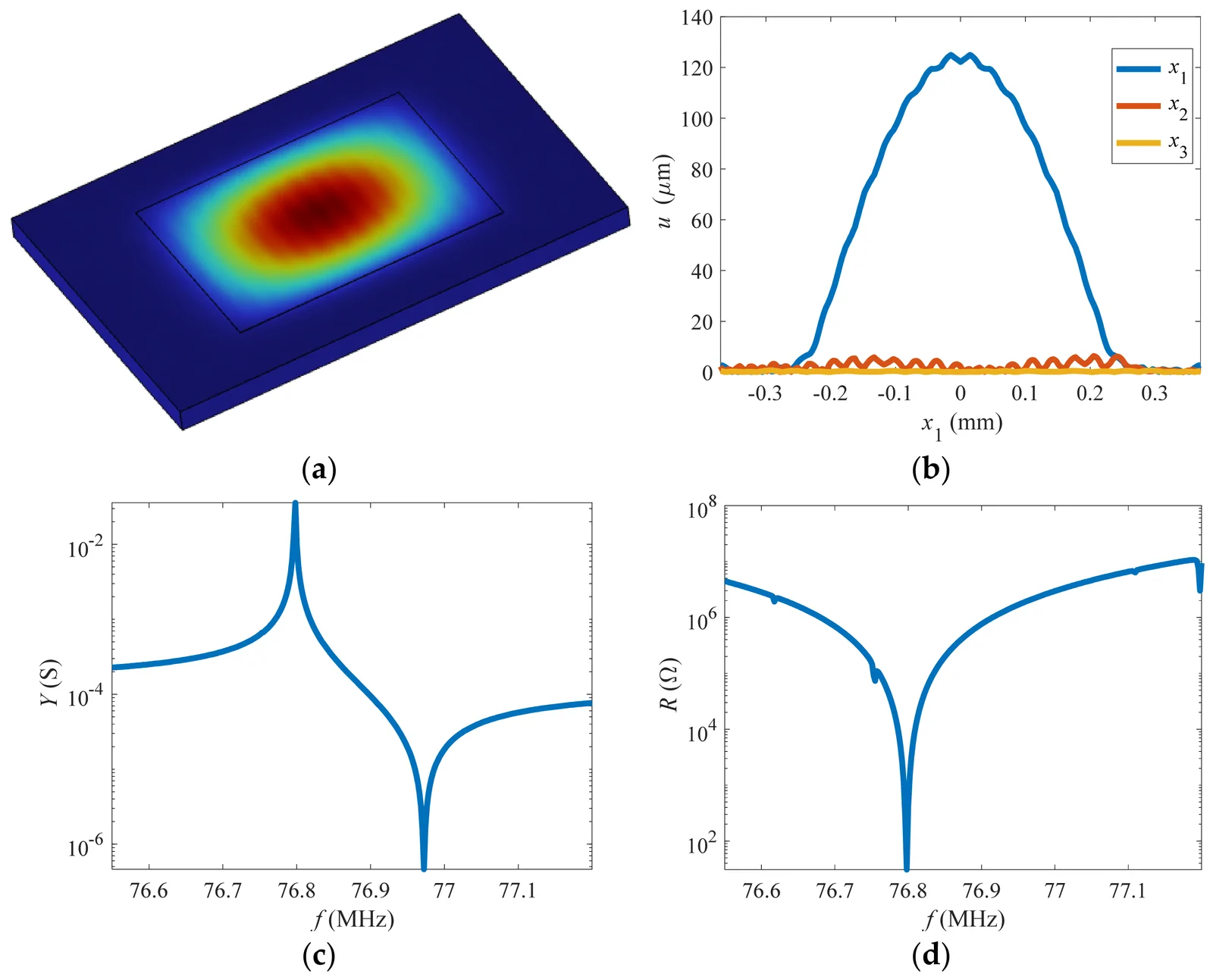
Results of the solid electrode model: (**a**) 3D vibration mode; (**b**) Distribution of displacement components at 76.798 MHz; (**c**) Admittance versus frequency; (**d**) Resistance versus frequency.

The numerical results of the solid electrode model are shown in Figure 2. The three-dimensional mode shape demonstrates localized thickness-shear vibration concentrated in the electrode-covered region. Hence vibration energy is effectively trapped within the central area, with almost no escape to the periphery of the wafer. The displacement amplitude follows an approximately Gaussian distribution on the wafer plane, reaching its peak at the electrode center and decaying rapidly toward the electrode edge, while the displacement near the wafer boundary approaches zero. This indicates that the mass loading effect of the electrodes effectively constrains vibration energy and suppresses edge leakage. The displacement *u*_2_ in the thickness direction is much smaller than the thickness shear displacement *u*_1_, and the displacement *u*_3_ in the width direction is almost zero. Therefore, the coupling between the thickness-shear mode and the bending mode or other parasitic modes is relatively small.

At the first thickness-shear frequency, the dominant displacement component corresponds to thickness-shear, presenting a symmetric single-peak distribution along the centerline of the wafer. The minor displacement components, induced mainly by interfacial stress disturbances near the electrode edges, exhibit amplitudes below 5% of the primary component and do not cause significant interference with the fundamental mode. The smooth displacement distribution also verifies that the mesh resolution is sufficient to capture the electrode edge effects.

The curves of admittance and resistance versus frequency both exhibit typical resonance characteristics. A single prominent admittance peak appears at the resonance frequency, where the mechanical vibration amplitude and the current reach their maxima. The admittance decreases rapidly when deviating from the resonant state. Over the full frequency range examined, only one major peak is observed without spurious fluctuations, indicating no excitation of parasitic modes. The symmetric admittance peak reveals uniform crystal loss and electrode damping in the frequency domain, free from abnormal nonlinear effects. Meanwhile, the resistance reaches its minimum at the resonance frequency, which is the typical feature that reactance vanishes and only dynamic resistance remains at resonance. These results confirm that the solid electrode model stably characterizes the response of the fundamental thickness-shear mode and closely approximates the physical behavior of actual devices.

### 2.4. Thin-Film Electrode Model

The thin-film electrode model simplifies the electrodes as ideal conductive films, neglecting their thickness, mass, and stiffness, while retaining the electrical boundary effect. Electrical coupling constraints are employed to apply signals in this model, but no mechanical loading effect is introduced. Since it does not require the construction of solid electrode meshes, this approach is computationally efficient and suitable for rapid estimation of the influence of electrical boundary conditions on the model response. However, it fails to reflect the effects of actual electrode mass and stiffness on the vibration behavior of the resonator.

**Figure 3:**
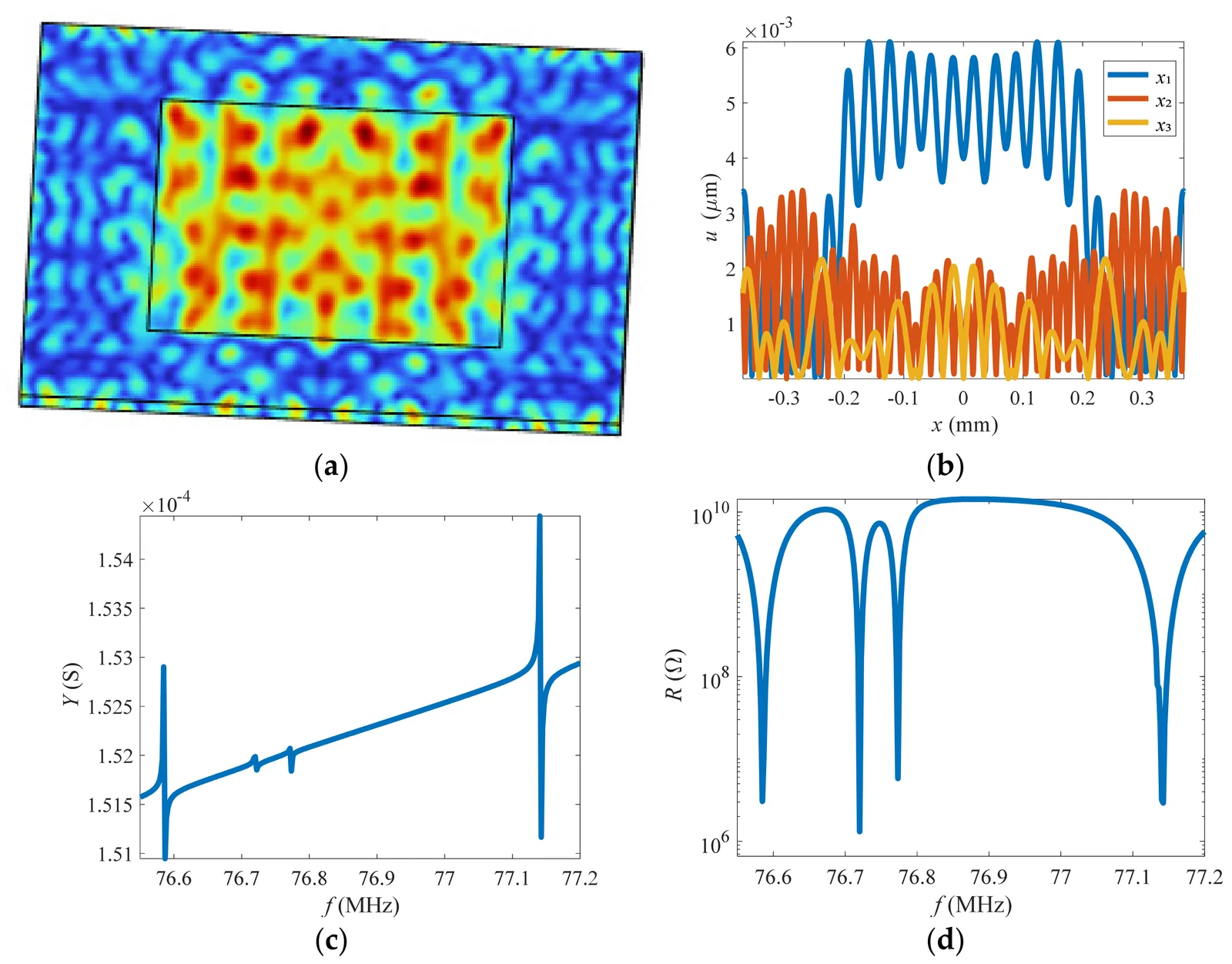
Results of the thin-film electrode model: (**a**) 3D vibration mode; (**b**) Displacement components at 76.79 MHz; (**c**) Admittance versus frequency; (**d**) Resistance versus frequency.

Figure 3 illustrates the numerical results of the thin-film electrode model. From (a) and (b), it can be observed that this model fails to produce a well-defined thickness-shear mode shape. The displacement distribution lacks central localization features, which indicates the fundamental thickness-shear mode is not effectively excited. From (c) and (d), the curves of corresponding admittance and resistance versus frequency exhibit much more resonance frequencies near the theoretical value. These observations indicate that, after neglecting the electrode mass and stiffness, the model is unsuitable for describing the actual operating state of thickness-shear resonators, even though the thickness ratio 2*H*/*H_e_* of the quartz plate to the electrode is as large as 72.8.

### 2.5. Added Mass Electrode Model and Combinating Electrode Model

The added mass electrode model equates the geometric thickness and mass of electrodes to surface mass loading. The added mass is defined as the mass per unit area of electrodes, which is equal to the electrode density multiplied by its thickness. Distributed surface loads are applied on the electrode-covered areas of the upper and lower wafer surfaces. This approach preserves the dominant influences of electrode mass on resonance frequency and energy trapping, while reducing modeling complexity and computational cost by omitting the mesh division of solid electrodes and interfacial constraints. Accordingly, the added mass electrode model is well-suited for rapidly evaluating thickness-shear vibration characteristics.

Besides, the combining electrode model of added mass with thin-film introduces an equivalent mass loading on the basis of the electrical boundary conditions of the thin-film electrode model, thereby accounting for the effects of electrodes on electric field distribution as well as local inertial effect. This model compensates for the frequency deviations caused by the neglecting electrode mass in the pure thin-film electrode model, while avoiding the complex three-dimensional electrode meshing and interface constraint handling required in the solid electrode model. As a result, it achieves a favorable balance between computational efficiency and physical completeness.

**Figure 4:**
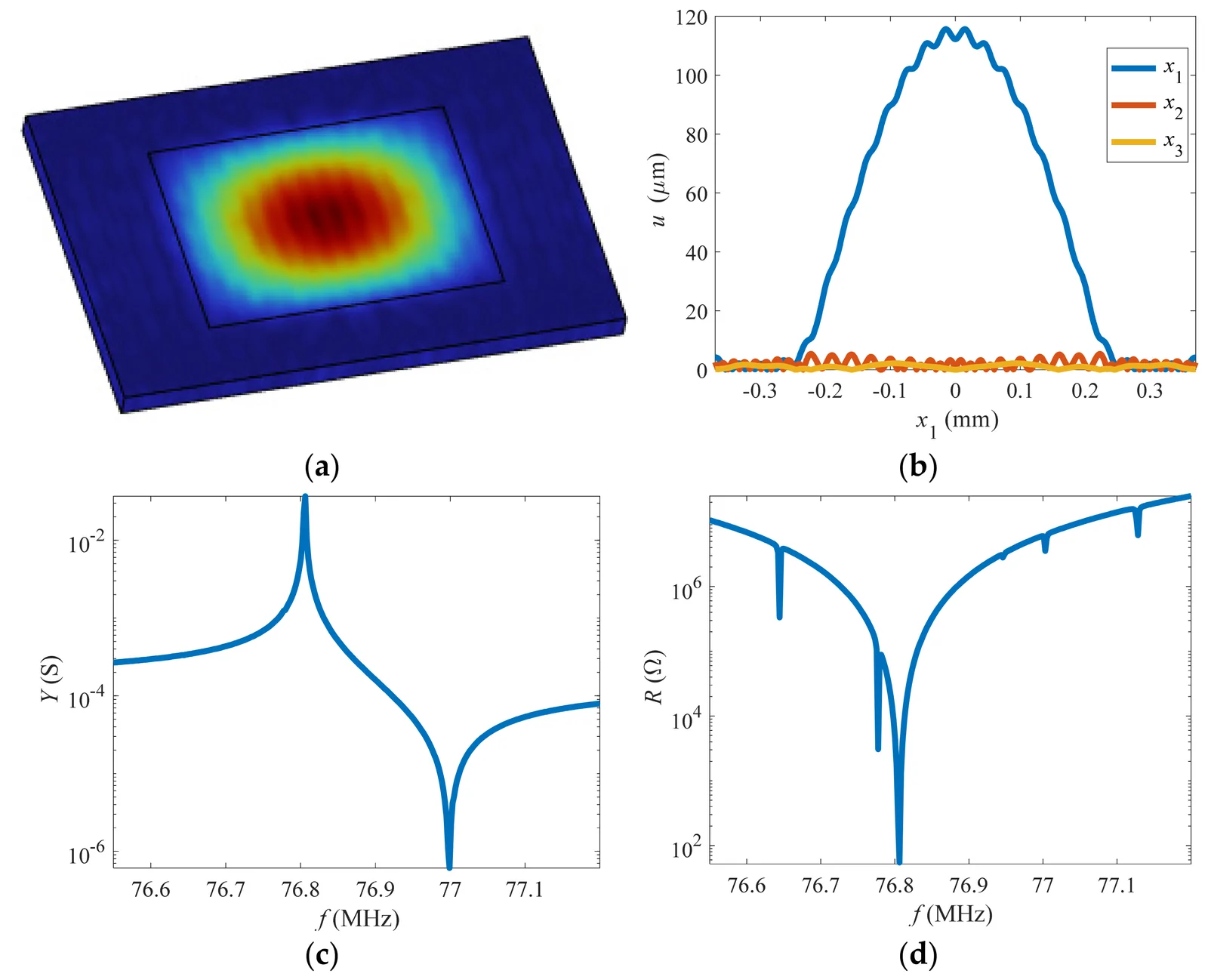
Results of the added mass electrode model: (**a**) 3D vibration mode; (**b**) Displacement components at 76.806 MHz; (**c**) Admittance versus frequency; (**d**) Resistance versus frequency.

**Figure 5:**
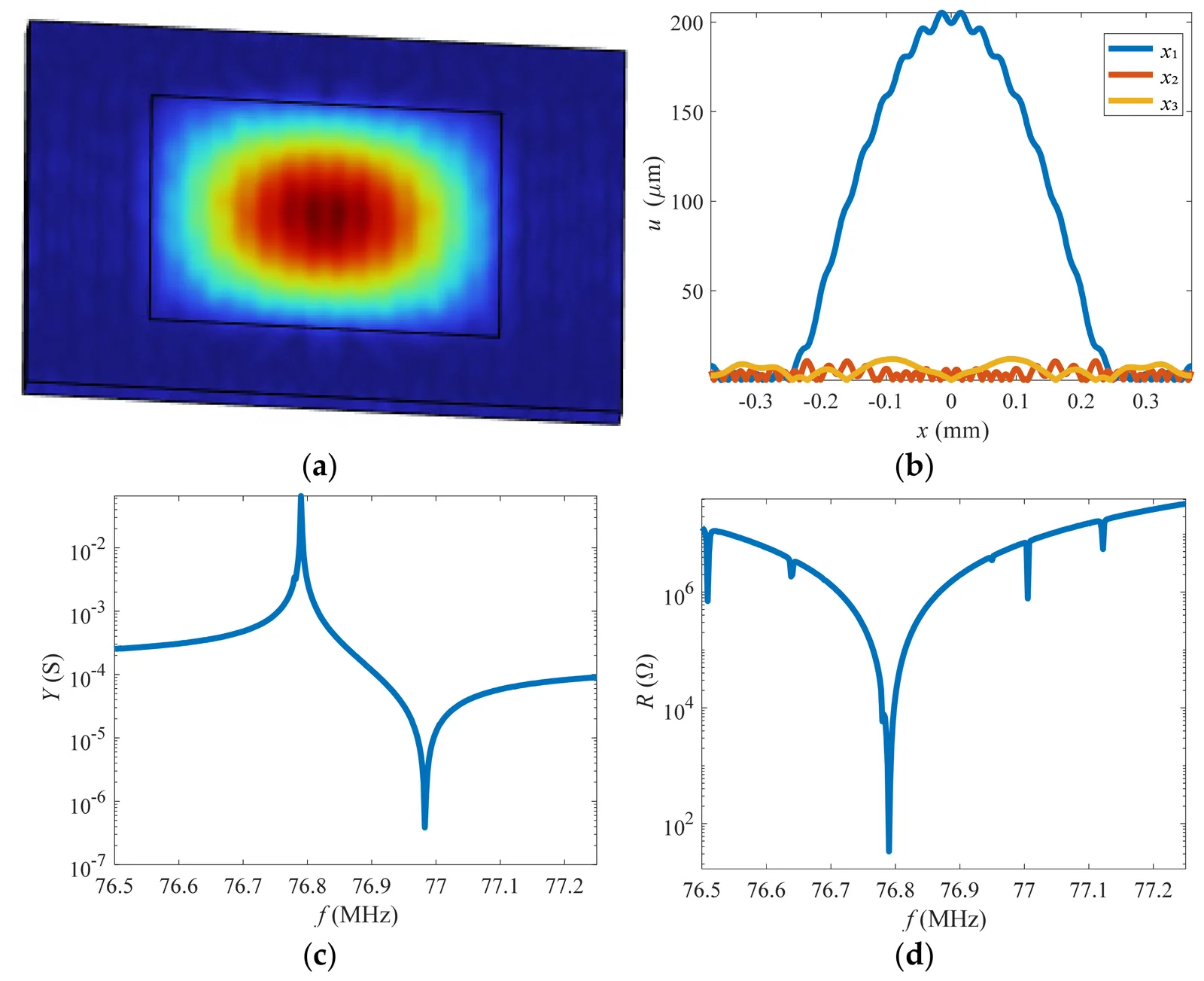
Results of the combining electrode model of added mass with thin-film: (**a**) 3D vibration mode; (**b**) Displacement components at 76.79 MHz; (**c**) Admittance versus frequency; (**d**) Resistance versus frequency.

Numerical results of the added mass electrode model as shown in Figure 4, and the combining electrode model of thin film with added mass as shown in Figure 5, both demonstrate trends that corroborate the predictions of the solid electrode model. Nevertheless, in contrast to the solid electrode model, the added mass electrode model neglects the stiffness, the geometric confinement and thickness of the electrode. This omission leads to an overestimation of the resonance frequency from 76.798 to 76.806 MHz. Although this effect can be ignored for thin electrodes, inherent estimation deviations persist in the energy trapping distribution and electromechanical impedance. Crucially, these discrepancies scale monotonically with increasing electrode thickness. However, for ultra-thin electrodes, these errors will be smaller. The added mass model is suitable for the rapid calculating resonance frequency and vibration properties. In contrast, the combining electrode model of added mass with thin-film introduces additional elastic stiffness and continuously distributed inertial loads. The interfacial coupling of the thin film highly localizes vibrational energy within electrode-covered zones. The thin-film model merely considers in-plane elasticity while ignoring all stresses in the thickness direction inside electrodes, thus providing a weaker stiffness contribution than the three-dimensional solid electrode model. Accordingly, the amplitudes of thickness-shear displacement components increase significantly under the same excitation. But this phenomenon gradually weakens as electrode thickness decreases.

In summary, the added mass electrode model enables fast and relatively accurate numerical simulations for the quartz resonator equipped with ultra-thin electrodes. The combining electrode model of thin-film with added mass takes both the frequency shift induced by electrode mass and the influences of thin-film electrodes on electric field distribution into account, yet it introduces extra mechanical constraints. Consequently, the added mass electrode model delivers reliable simulation results with satisfactory accuracy, whereas the combining electrode model of thin-film with added mass exhibits lower computational accuracy compared with the added mass electrode model.

### 2.6. Comparison of Model Results and Computational Efficiency

**Figure 6:**
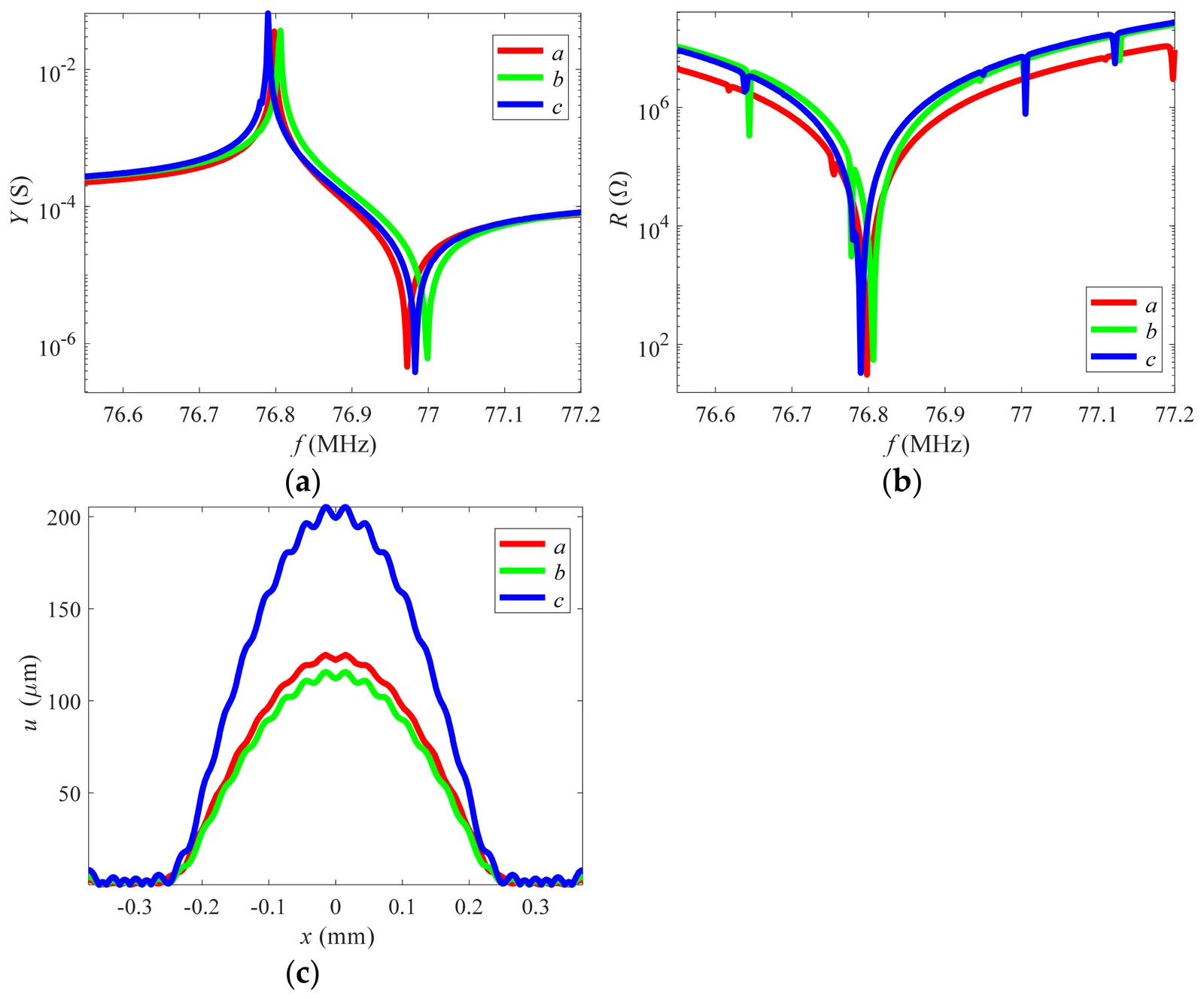
Comparison of results from three models. The curves a, b, and c represent the solid electrode model, the added mass electrode model, and the combining electrode model, respectively. (**a**) Admittance versus frequency; (**b**) Resistance versus frequency; (**c**) Comparison of primary displacement *u*_1_.

Figure 6 compares the frequency response of admittance and resistance, and primary displacement distribution of the solid electrode model, added mass electrode model and combining electrode model.

All three models produce distinct admittance peaks and resistance valleys near 76.8 MHz with minor frequency deviations, which means that all electrode models can capture the primary resonance characteristics accurately. The resistance curves further confirm that the thickness-shear primary mode is fully excited for all models. The solid electrode model and added mass electrode model yield highly consistent admittance and resistance curves, and displacement distributions, which proves that equating electrodes to equivalent mass can well reproduce the resistance variation near the primary resonance frequency. The combining model shows more obvious resistance disturbance in some frequency points, indicating that the stiffness and mass distribution of thin-film alter the equivalent dynamic boundary conditions in the electric field loading region and further affect the details of frequency response.

The primary displacement distributions of these three models all follow the rule that the amplitude is the maximum in the central electrode region and decays towards the edge, which reflects the energy trapping feature of thickness-shear vibration localized in the electrode area. The central displacement amplitude of the combining model is approximately twice that of the solid electrode model and added mass model, revealing that the coupling effect of mass and stiffness is amplified in the coupling model, leading to more concentrated displacement response in the electrode area.

The solid electrode model can fully characterize the electrode structure, but it requires a large number of degrees of freedom and mesh elements as listed in Table 2. The combining model of thin-film with added mass achieves high response accuracy. However, the added mass model possesses excellent computational efficiency and acceptable accuracy. Therefore, it is recommended as the dominant simulation model for the preliminary geometric design and structural optimization of quartz resonators.

The *Q* factors from the Solid model, thin film electrode model, added mass model, and combined thin film with added mass model are further shown in Table 3. All four models use the same complex stiffness matrix and quartz viscosity coefficients, yet the predicted *Q* factors differ significantly. This shows that how the electrode geometry and mechanical properties are treated has a major impact on *Q* factor prediction. The solid model accounts for the electrode’s geometry, thickness, mass, and stiffness. The thin film electrode model ignores all these mechanical properties. The added mass model and the combined model include the electrode mass but simplify its three-dimensional geometry and stiffness. As a result, the four modeling approaches give different complex eigenfrequencies and *Q* factors. Notably, the *Q* factors predicted by the added mass model are closer to the solid model. Owing to the omission of dielectric loss, the calculated *Q*-factor is larger than the experimental results.

## 3. Influences of Cut Angle and Added Mass on Overall Performance

### 3.1. Effects of Cut Angle on Frequency and Resistance

The material parameters of quartz in the load coordinate system are dependent upon the cut angle. Thus, the vibration characteristics of quartz resonators vary significantly with cut angle. Subsequently, the added mass electrode model is adopted to explore the effect of cut angle on the first order natural frequency and electric parameters.

**Figure 7:**
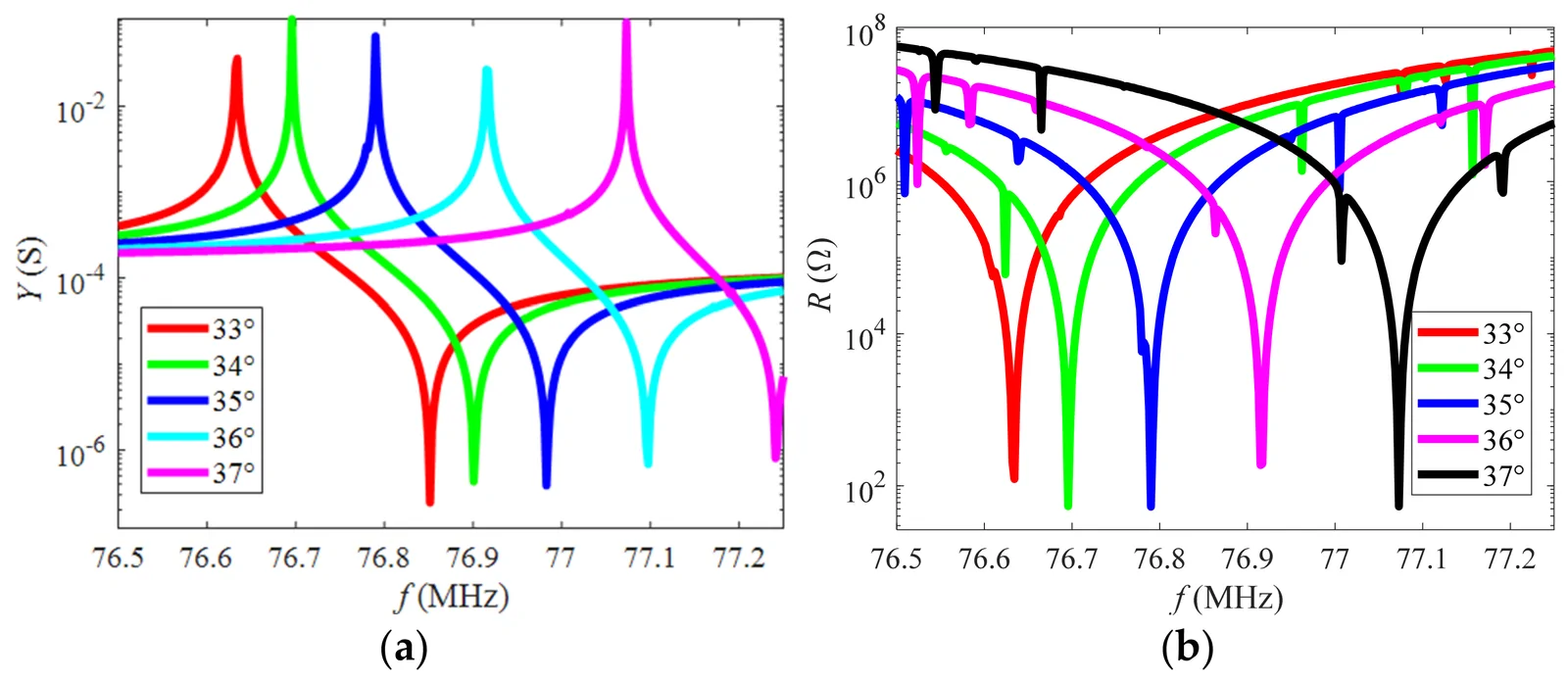
Electric parameters versus frequency for different cut angles calculated by the added mass electrode model: (**a**) Admittance; (**b**) Resistance.

Figure 7 presents the frequency dependent curves of admittance and resistance at different cut angles. The cutting angle ϕ is taken around 35.25° of the AT cut. As the cut angle increases, the resonant peaks shift towards higher frequencies, and the valleys on the resistance curves also shift accordingly. This indicates that the cut angle directly changes the natural frequency of the resonator. The overall profile of the curves remains stable with cut angle variation, which indicating that the fundamental mode type does not change. The increase of cut angle alters the equivalent shear stiffness of quartz and further induces resonance frequency shift.

**Figure 8:**
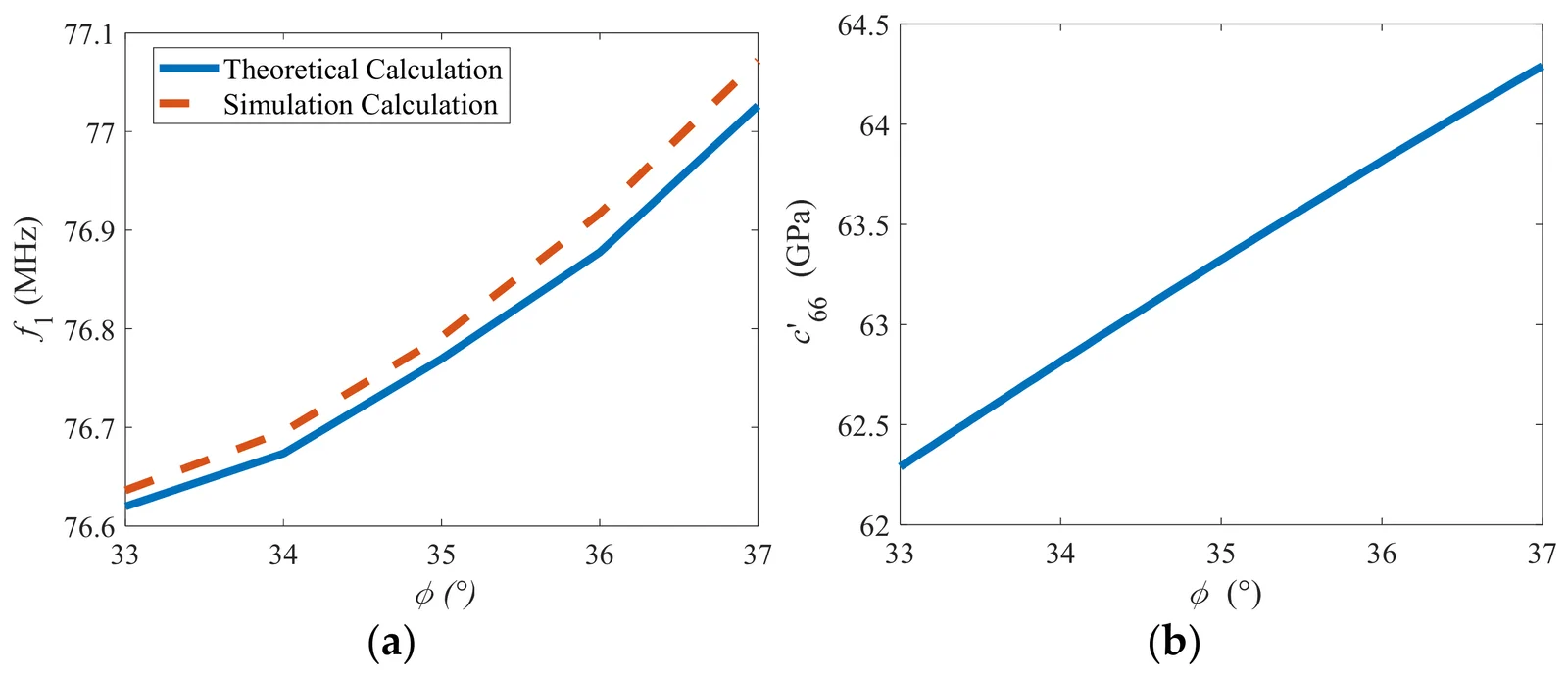
Thickness-shear resonance frequency and transformed shear stiffness coefficient versus cut angle: (**a**) First resonance frequency calculated by theory and numerical simulation with added mass electrode model; (**b**) Transformed shear stiffness coefficient.

In order to analyze the mechanism by which the tangent angle of a quartz resonator changes the resonance frequency, Figure 8 is plotted to show the solutions of the first resonance frequency calculated by theory and numerical simulation with added mass electrode model. Since the transformed shear stiffness coefficient as a function of the tangent angle, despite using a one-dimensional theoretical model, the theoretical results have the same change trend as numerical results. As the cut angle increases, the transformed shear stiffness coefficient increases, the structure becomes rigid, and therefore the first thickness-shear resonance frequency also increases.

### 3.2. Effects of Added Mass on Frequency and Resistance

**Figure 9:**
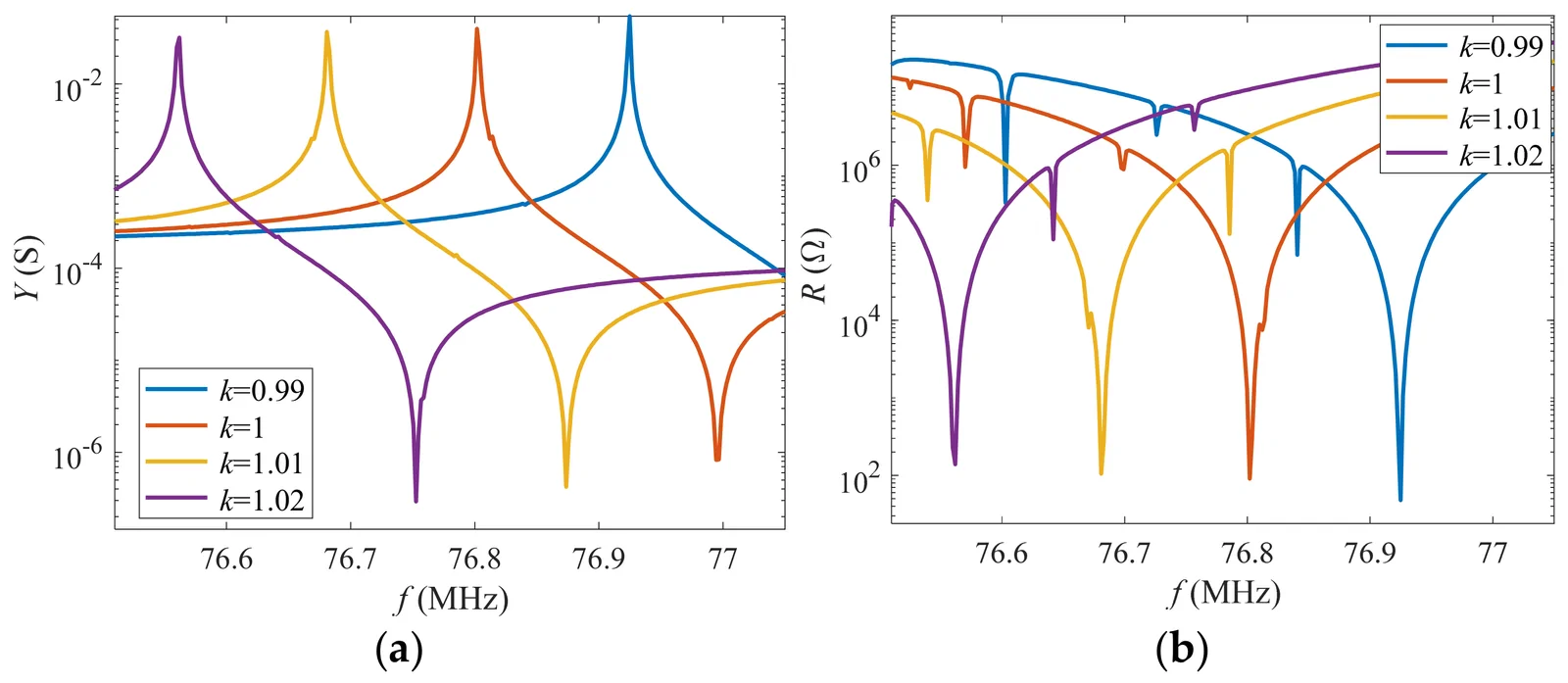
Electric parameters versus frequency for different mass coefficients: (**a**) Admittance; (**b**) Resistance.

A mass coefficient *k* is introduced to quantify the relative variation of electrode mass, with the original electrode mass as the benchmark. The mass coefficient *k* ranges from 0.99 to 1.02 to investigate the influences of electrode density and mass on the model outputs. As shown in Figure 9, the admittance peaks shift towards lower frequencies with the increase of *k*, which means that the rise of added mass reduces the resonance frequency. The resistance valleys move synchronously to low frequency regions, consistent with the variation law of admittance peaks. Compared with the amplitude variation of curves, the shift of resonant positions is more remarkable, which reveals that added mass affects the frequency response mainly by changing the equivalent inertia of the system, i.e., the typical mass loading effect. The increase in electrode mass increases the structural inertia, resulting in a decrease in resonance frequency, which explains the left shift of the resistance valley. Even small changes in *k* within the range of 0.99~1.02 can cause significant resonance frequency shifts, indicating that additional mass is a key factor determining the calculation accuracy of high-frequency quartz resonators.

In addition, the increase in electrode mass leads to a corresponding increase in resistance. Near the resonance frequency, the energy conversion between mechanical vibration and electric field reaches the maximum, and the motional branch dominates the equivalent circuit, corresponding to the resistance valley. Near the anti-resonance frequency, phase cancellation occurs between the motional branch and static capacitance branch, resulting in resistance rise. Meanwhile, the resistance characteristics are also closely related to the cut angle. The cut angle changes the piezoelectric coupling strength and equivalent elastic parameters, so it affects the resonance and anti-resonance frequencies, and resistance valleys simultaneously. In practical design, cut angle deviation will lead to frequency drift and resistance mismatch. Therefore, as a key structural parameter affecting frequency stability, the cut angle must be strictly controlled.

In summary, the cut angle and added mass affect the thickness-shear resonance frequency and resistance of the resonator through different mechanisms: the cut angle alters the anisotropic material parameters and piezoelectric coupling characteristics, while the added mass changes the equivalent inertia of the resonator. Both factors will lead to the shift of resonance frequency and resistance valleys. Hence, in the modeling and design of the quartz resonator, the comprehensive effects of cut angle deviation and electrode mass must be considered to improve the prediction accuracy of resonance frequency and resistance.

## 4. Conclusions

Based on the systematic finite-element comparative analysis of four electrode models for the 76.8 MHz AT-cut quartz resonator, the following conclusions are drawn:

(1) The solid electrode model provides the computational fidelity by comprehensively considering electrode stiffness and mass, and energy trapping. Conversely, the pure thin-film model ignores mechanical mass and stiffness of the electrode, resulting in significant errors that render it unsuitable for accurate device characterization.
(2) The added mass model provides a superior trade-off between computational accuracy and cost. It preserves the mass loading effect of the electrode while ignoring its stiffness contribution, making it an ideal model for rapid parametric research.
(3) Cut angle and electrode mass have a significant impact on the fundamental frequency of thickness-shear mode. The cut angle governs the effective shear modulus via anisotropic elasticity, while the electrode mass affects the vibrational inertia.

This work enhances the theoretical foundation for numerical simulation and structural optimization, paving the way for improved design reliability and manufacturing yield in high-frequency resonators.

## Figures and Tables

**Table 1:** Geometric dimensions of the 76.8 MHz quartz resonator.

Parameter	Symbol	Value
quartz plate length	*L*	0.74 mm
quartz plate width	*W*	0.493 mm
quartz plate thickness	2*H*	0.0182 mm
electrode length	*L_e_*	0.435 mm
electrode width	*W_e_*	0.306 mm
electrode thickness	*H_e_*	250 nm

**Table 2:** Computational efficiency of three electrode models.

Electrode Model	Degrees of Freedom	Computation Time
Solid model	429,254	2 h 37 min 14 s
Added mass model	388,410	1 h 52 min 28 s
Combining model of thin-film with added mass	388,410	2 h 42 min 04 s

**Table 3:** *Q* factors calculated from the complex eigenfrequencies of the four electrode models.

Electrode Model	*Q* Factor
Solid model	187,217
Added mass model	187,943
Thin-film electrode model	165,524
Combining model of thin-film with added mass	186,191

## Data Availability

The data presented in this study are available on request from the corresponding author. The raw experimental measurements from our industrial partner are restricted under a non-disclosure agreement.
